# TMO-Net+: An Enhanced Tumor Multi-Omics Pre-Trained Network for Multi-Task Learning in Oncology

**DOI:** 10.3390/genes17091085

**Published:** 2026-09-09

**Authors:** Wei Liu, Xuan Liu, Shuyu Zhou, Kaiyang Li, Xiangzhi Wang, Ke Chen, Lilu Guo, Rui Zhang, Qingzhi Su

**Affiliations:** 1 School of Computer and Software, Nanyang Institute of Technology, Changjiang Road 80, Nanyang 473000, China; xuanliunit@nyist.edu.cn (X.L.); shuyuzhou@nyist.edu.cn (S.Z.); kaiyangli@nyist.edu.cn (K.L.); xiangzhiwang@nyist.edu.cn (X.W.); kechen11neu@gmail.com (K.C.); 2 Academy for Electronic Information Discipline Studies, Nanyang Institute of Technology, Changjiang Road 80, Nanyang 473000, China; guolilu@stu.xidian.edu.cn; 3 Nanyang Central Hospital, Gongnong Road 312, Nanyang 473000, China; nyszxyyzr@163.com; 4 Xinye County Xinruo Electronic Technology Co., Ltd., No. 009 Xingye Road, Xinye County, Nanyang 473000, China; qzhisu@163.com

**Keywords:** tumor prediction, feature attention encoder, gated Mixture-of-Experts, classifier loss, TMO-Net+

## Abstract

Background: Tumor heterogeneity arises from complex interactions among diverse biological factors, posing a major challenge for the development of robust multi-omics data integration methods. While the existing Tumor Multi-Omics pre-trained Network (TMO-Net) enables the fusion of multi-omics features into unified representations, its practical utility is constrained by issues such as missing modalities, incomplete within-omics data, and high-dimensional noise. To overcome these limitations, we propose TMO-Net+, an enhanced architecture specifically designed to improve the robustness and reliability of multi-omics modeling. Methods: TMO-Net+ introduces several coordinated architectural enhancements. First, a feature attention encoder is applied to each omics data type to reduce the influence of modality-dependent input variation. Second, we combine a gated Mixture-of-Experts (MoE) module with a Product-of Experts (PoE) mechanism to capture sample-specific contributions and enable robust inference even when partial omics data are available. Additionally, a supervised deep classification head with a tailored loss function is incorporated to enhance the separability of learned embeddings in the latent space. Results: Extensive experiments on pan-cancer datasets demonstrate that TMO-Net+ consistently outperforms the original TMO-Net, as measured by LogME scores. Furthermore, in various downstream tasks (e.g., pan-cancer classification, primary/metastatic site prediction, and prognostic modeling), TMO-Net+ achieves superior performance under partial-omics settings, which proves that it enhances the robustness and cross-cancer transferability of the multi-omics representations. Conclusions: The proposed TMO-Net+ improves the robustness and cross-cancer transferability of multi-omics representations within the evaluated TCGA cohorts. Biological interpretability analyses further show that TMO-Net+ prioritizes established cancer-driver genes, preserves cancer-dependent molecular-state information, and adaptively redistributes relative modality contributions across molecular states. By addressing modality-level missingness and modality-dependent input variation, it offers a reliable framework for integrative tumor analysis within the evaluated TCGA cohorts.

## 1. Introduction

Tumorigenesis and progression are systemic processes unfolding across diverse biological scales and organizational levels. These processes are driven by a complex interplay of genomic alterations, epigenetic modifications, transcriptional dysregulation, and copy-number variations (CNVs), which collectively engender profound heterogeneity in both molecular landscapes and clinical phenotypes [1,2,3]. The advent of high-throughput sequencing, coupled with large-scale repositories such as The Cancer Genome Atlas (TCGA) and the International Cancer Genome Consortium (ICGC), has revolutionized the characterization of these molecular intricacies [4,5,6]. Increasing evidence suggests that integrating multi-omics data provides superior modeling capacity compared to single-omics approaches, offering deeper insights into oncogenic mechanisms and more robust support for clinical decision-making [7,8,9,10]. Consequently, multi-omics representation learning has emerged as the cornerstone of translational oncology, including pan-cancer stratification, primary-versus-metastatic classification, and prognostic risk assessment [11].

Despite the great potential of the above-mentioned multi-omics representation learning, its clinical analysis is hindered by substantial computational challenges. The first major challenge is the curse of dimensionality coupled with cross-omics heterogeneity: the feature dimensionality typically far exceeds the sample size, and different omics (e.g., continuous gene expression versus sparse mutation profiles) follow markedly different distributions and noise patterns [12]. This high-dimensional, low-sample regime often leads to overfitting and unstable representations. Consequently, dimensionality-reduction and regularization strategies including manifold-regularized learning have been explored to improve generalization [13,14]. The second pervasive challenge is missingness [15], which occurs both at the modality level (missing omics types for some patients) and within individual omics (missing values/features due to measurement limitations). Due to constraints in tissue quality, sequencing costs, or platform discrepancies, individual patients often lack complete omics profiles, making unpaired observations the norm rather than the exception [16]. Many existing methods for case analysis implicitly assume complete omics data and thus resort to complete-case analysis by discarding samples with missing omics data. This practice not only wastes valuable clinical data and reduces statistical efficiency, but may also introduce estimation and selection bias when missingness is not completely at random [16,17]. Consequently, developing a unified multi-omics integration framework that is both resilient to high-dimensional noise and capable of accommodating multi-task demands under missing omics data remains a critical step forward.

Currently, various multi-omics integration strategies have been proposed, but there are still significant limitations. Early statistical methods [18], such as iCluster [19], similarity network fusion (SNF) [20], and MOFA+ [21], have demonstrated good performance in dimensionality reduction and clustering, but are often insufficient for capturing complex nonlinear interactions. More recently, deep learning has emerged as a dominant paradigm [22]. OmiVAE [23] and XOmiVAE [24] learn latent representations via autoencoder-style reconstruction. MOGONET [25] leverages graph convolution to enhance classification. DeepSurv [26], Cox-nnet [27], and SALMON [28] extend neural modeling to survival analysis. To tackle more challenges, researchers have introduced contrastive learning, meta-learning, and generative modeling. For example, PCLSurv [29] improves survival prediction via cross-view contrastive objectives. MMOSurv [30] applies meta-learning to few-shot cancer types. DeepGAMI [31] and joint VAEs [32] attempt modality completion through auxiliary learning or joint distributions. CMOT [33] aligns modalities using optimal transport theory. Nevertheless, these advanced methods share several shortcomings. Some contrastive frameworks require carefully constructed positive relationships (e.g., cross-omics pairing within the same sample or prototype/label-level similarity), and therefore depend heavily on paired multi-omics observations. Under missing or unpaired modalities, additional objectives and architectural designs are typically required for robustness [29,34]. Furthermore, for specific tasks such as metastasis prediction, existing models (e.g., MetaCancer [35]) may still offer limited interpretability for deep multimodal interactions. Finally, many fusion strategies (e.g., HFBSurv [36] and PathCNN [37]) often adopt predefined fusion designs (e.g., concatenation or fixed hierarchical/bilinear modules), and may not explicitly model sample-wise dynamic gating or provide a clear decomposition of modality contributions, which can be critical when modality quality is imbalanced or modalities are missing [38]. Motivated by the success of foundational models and large-scale biomedical pre-training [39,40], TMO-Net [41] overcomes the aforementioned challenges to some extent by learning a general multi-omics representation. However, it still faces challenges arising from modality-level missingness, within-omics incompleteness, and high-dimensional noise in practical issues.

**Figure 1:**
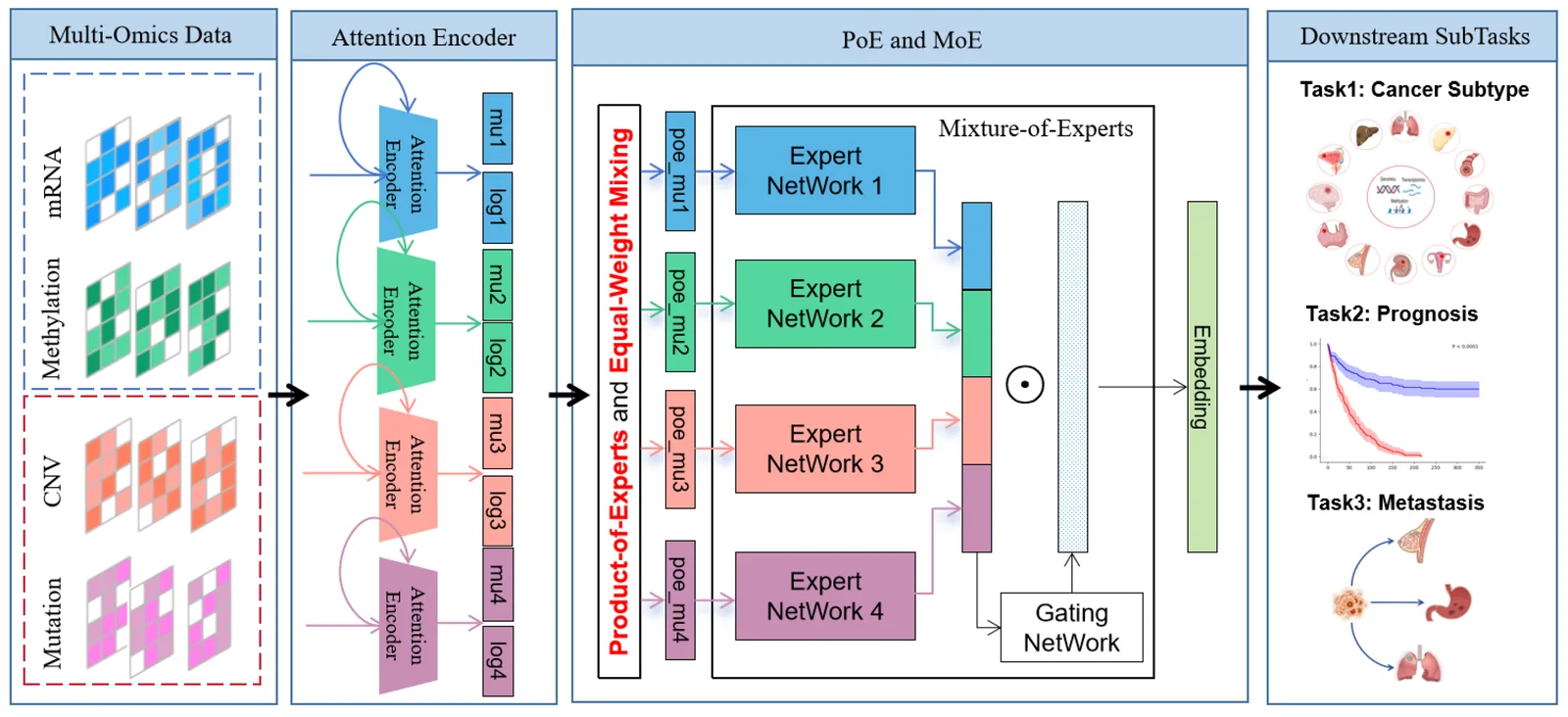
Workflow of the proposed enhanced Tumor Multi-Omics pre-trained Network (TMO-Net+). The left side illustrates the foundation-model pre-training stage using four TCGA omics modalities: RNA-seq gene expression, DNA methylation, somatic mutation, and copy-number variation (CNV). Each modality is processed by a dedicated feature-attention encoder, which recalibrates the hidden representation using a sigmoid attention mask and estimates the mean and log-variance of the corresponding latent distribution. The Product-of-Experts (PoE) module integrates the posterior distributions of the available modalities to obtain a fused latent posterior. For an observed modality, its modality-specific latent mean is equally combined with the fused PoE mean, whereas the fused PoE mean is used as the latent representation when that modality is missing. The resulting modality-wise representations are transformed by four expert networks, and a centralized gating network generates normalized, sample-specific modality weights to construct the joint embedding. During supervised pre-training, the classification head, contrastive loss, and classification loss promote cancer-type separability of the learned representation. The right side shows the transfer of the pretrained encoder and fusion modules to three downstream tasks: pan-cancer classification, primary/metastatic site prediction, and prognostic modeling.

To address the aforementioned issues, we propose an enhanced Tumor Multi-Omics pre-trained Network, termed TMO-Net+, for improved representation learning from multi-omics data. The enhancement integrates an attention mechanism to build a feature attention encoder and introduces a gated MoE after the PoE structure used in the original TMO-Net. As illustrated in Figure 1, during the pre-training stage, modality-specific feature attention encoders, PoE aggregation, and gated MoE fusion are jointly employed to learn an integrated multi-omics representation. The pretrained model is subsequently transferred and fine-tuned for pan-cancer classification, primary/metastatic prediction, and prognostic modeling. Experiments conducted on pan-cancer datasets that cover four TCGA omics modalities, specifically gene expression, DNA methylation, somatic mutations, and copy-number variation (CNV), demonstrate that TMO-Net+ significantly surpasses the original TMO-Net in LogME scores.Furthermore, across all three downstream tasks, TMO-Net+ consistently achieves improved overall performance, while controlled modality-removal analyses demonstrate retained representation stability under partial-omics conditions, supporting its robustness and cross-cancer transferability within the evaluated TCGA cohorts. Biological interpretability analyses further show that TMO-Net+ prioritizes established cancer-driver genes and depends on molecular feature identities. The learned joint representations also retain cancer-dependent molecular-state information, while the sample-specific MoE weights reveal relative changes in modality contributions across molecular states.

Our main contributions can be summarized as follows:

(1) We design a feature attention encoder, which incorporates an attention mechanism to reduce the influence of modality-dependent input variation in each omics modality.
(2) We propose TMO-Net+, an enhanced tumor multi-omics pre-trained network. It consists of a feature attention encoder, MoE, a supervised deep classification head and the corresponding loss function, all of which are newly incorporated into the TMO-Net framework.
(3) We assess the capability of the proposed TMO-Net+ model on pan-cancer datasets that cover four omics modalities, which are gene expression, DNA methylation, somatic mutations, and copy-number variation. Extensive experiments indicate that TMO-Net+ achieves the highest mean LogME score among the evaluated variants and improved overall performance across the three downstream tasks. involving pan-cancer classification, primary/metastatic site prediction, and prognostic modeling.

## 2. Methods

### 2.1. Feature Attention Encoder

The feature attention encoder aims to extract latent feature representations from high-dimensional multi-omics data. Specifically, it utilizes a hierarchical linear layer architecture with a gated attention mechanism to adaptively prioritize information while reducing the influence of modality-dependent input variation and redundancy.

#### 2.1.1. Hierarchical Linear Layer

The encoder is constructed using a multilayer perceptron (MLP). For a given omics input $x^{m}\in R^{D_{m}}$, the hidden representations are extracted through a series of linear transformations:

(1) $$h_{k}^{m}=\sigma \;\left(\mathrm{BN}\;\left(W_{k-1}^{m}h_{k-1}^{m}+b_{k-1}^{m}\right)\right)$$

where $h_{k}^{m}$ represents the feature vector of the *k*-th hidden layer within the MLP for each omics modality *m*, with the initial state defined as $h_{0}^{m}=x^{m}$. Here, BN(·) denotes Batch Normalization, and σ(·) signifies a non-linear activation function (e.g., ReLU). The terms $W_{k}^{m}$ and $b_{k}^{m}$ denote the learnable weights and biases of the *k*-th linear layer, respectively.

#### 2.1.2. Gated Feature Attention Mechanism

A key component of our architecture is the incorporation of a gated feature attention mechanism, designed to bridge the raw input space with the deep semantic features. Unlike standard feed-forward networks, our model employs a global skip-connection to recalibrate the final hidden features based on the original omics input.

Specifically, an identity projection ϕ(·) is applied to the original omics input $x^{m}$ to align its dimensionality with the final hidden layer $h_{L}^{m}$. An attention mask $g^{m}$ is then computed via a Sigmoid function:

(2) $$g^{m}=\phi \left(x^{m}\right)=\mathrm{Sigmoid}\;\left(W_{\mathrm{proj}}^{m}x^{m}+b_{\mathrm{proj}}^{m}\right)$$

The final attended feature $x_{\mathrm{att}}^{m}$ is obtained by performing an element-wise multiplication ⊙ between the deep features from the final *L*-th hidden layer and the gated attention mask:

(3) $$x_{\mathrm{att}}^{m}=h_{L}^{m}⊙g^{m}$$

This mechanism allows the model to selectively retain informative features while reducing the influence of modality-dependent input variation by utilizing the global context of the original omics input.

#### 2.1.3. Variational Inference and Reparameterization

To map the attended features into a probabilistic latent space, the encoder outputs the parameters of a multivariate Gaussian distribution. Two independent linear heads compute the mean vector ($\mu_{m}$) and the log-variance vector ($\log(\sigma_{m}^{2})$):

(4) $$\mu_{m}=W_{\mu ,m}x_{\mathrm{att}}^{m}+b_{\mu ,m},\;\log\left(\sigma_{m}^{2}\right)=W_{\sigma ,m}x_{\mathrm{att}}^{m}+b_{\sigma ,m}$$

To enable end-to-end training via standard backpropagation, we employ the reparameterization trick. The stochastic latent variable $z_{m}$ is sampled as:

(5) $$z_{m}=\mu_{m}+\epsilon ⊙\exp\;\left(\frac{1}{2}\log\left(\sigma_{m}^{2}\right)\right),\;\mathrm{where}\;\epsilon ∼N\left(0,I\right)$$

This ensures that the latent space is both expressive for clinical subtasks and mathematically tractable for optimization.

### 2.2. Cross Fusion Module with PoE and MoE

We present an enhanced cross-fusion module with PoE and MoE to learn comprehensive multi-omics representations from missing omics data. It unifies features from various attention encoders by projecting them into a common space.

#### 2.2.1. Product-of-Experts Method

To fuse multi-omics features extracted from distinct feature attention encoders, we adopted a PoE approach. Given a set of latent parameters ${\left\{\mu_{m},\log\left(\sigma_{m}^{2}\right)\right\}}_{m=1}^{A}$ predicted by *A* available single-omics encoders, the joint posterior distribution is modeled as a Gaussian distribution. The fusion process leverages the precision (inverse variance) of each encoder, effectively implementing a precision-weighted aggregation as follows:

(6) $$\sigma_{poe}^{2}={\left(\sum_{m=1}^{A}\exp\;\left(-\log(\sigma_{m}^{2})\right)\right)}^{-1}$$

(7) $$\mu_{poe}=\sigma_{poe}^{2}⊙\sum_{m=1}^{A}\left[\mu_{m}⊙\exp\;\left(-\log(\sigma_{m}^{2})\right)\right]$$

where ⊙ denotes element-wise multiplication. The parameters $\sigma_{poe}^{2}$ and $\mu_{poe}$ denote the shared variance and mean obtained by aggregating the posterior parameters of all available single-omics encoders.

This mechanism allows the model to produce a coherent latent representation by capturing the consensus among different input streams while naturally handling missing omics through the product formulation.

#### 2.2.2. Equal-Weight Mixing Strategy

To balance modality-specific self-reconstruction and cross-modal consensus, the mean representation ${\tilde{\mu }}_{m}$ is obtained via an equal-weight mixing strategy as follows:

(8) $${\tilde{\mu }}_{m}=\left\{\begin{array}{cc} \frac{1}{2}\left(\mu_{m}+\mu_{poe}\right),\; & \mathrm{if}\;\mathrm{omics}\;m\;\mathrm{is}\;\mathrm{present},\\ \mu_{poe},\; & \mathrm{if}\;\mathrm{omics}\;m\;\mathrm{is}\;\mathrm{missing}. \end{array}\right.$$

#### 2.2.3. Mixture-of-Experts Strategy

The MoE aims to adaptively perform a weighted fusion. Firstly, the expert networks in MoE facilitate the extraction of high-level features from each single-omics mean representation ${\tilde{\mu }}_{m}$. Each expert network $E_{m}$ consists of a linear transformation followed by a Gaussian Error Linear Unit (GELU) activation function. This can be formally defined as follows:

(9) $$h_{m}=E_{m}\left({\tilde{\mu }}_{m}\right)=\mathrm{GELU}\left(W_{m}{\tilde{\mu }}_{m}+b_{m}\right)$$

where $W_{m}\in R^{d\times d}$ and $b_{m}\in R^{d}$ are the learnable parameters of the *m*-th expert.

Secondly, a centralized Gating Network is utilized to observe the global context by concatenating the transformed expert features $H={\left[h_{1},h_{2},\ldots ,h_{M}\right]}^{T}\in R^{M\times d}$. These features are then flattened into a global context vector $g_{in}=\mathrm{vec}\left(H\right)\in R^{Md}$. The gating network *G* computes the omics contribution weights via a linear projection followed by a softmax function:

(10) $$\alpha =G\left(g_{in}\right)=\mathrm{Softmax}\left(W_{g}g_{in}+b_{g}\right)$$

where $\alpha ={\left[\alpha_{1},\alpha_{2},\ldots ,\alpha_{M}\right]}^{T}\in R^{M}$ denotes the sample-specific modality weight vector. The softmax operation normalizes the *M* modality weights for each sample, which satisfy

(11) $$\sum_{m=1}^{M}\alpha_{m}=1,\;\alpha_{m}\geq 0.$$

The parameters $W_{g}\in R^{M\times Md}$ and $b_{g}\in R^{M}$ are the learnable parameters of the gating network. Here, $\alpha_{m}$ represents the sample-specific relative contribution of the *m*-th modality.

Thirdly, each modality weight $\alpha_{m}$ is applied to the corresponding *d*-dimensional expert output. The weighted expert outputs are concatenated to form the joint embedding:

(12) $$O=\mathrm{vec}\left({\left[\alpha_{1}h_{1},\alpha_{2}h_{2},\ldots ,\alpha_{M}h_{M}\right]}^{T}\right)\in R^{Md}.$$

This design enables sample-specific modality weighting while preserving the modality-specific structure of the joint embedding, which may improve latent-space separability and downstream discriminative performance.

### 2.3. Loss Function

#### 2.3.1. Contrastive Loss

For the latent embedding O and the corresponding pan-cancer subtype-label vector y from the multi-omics datasets, we adopt a contrastive loss function [42] to enhance the discriminative capacity of the learned embeddings. First, we construct a pairwise distance matrix $D\in R^{N\times N}$. Each element $d_{ij}$ represents the distance between embeddings $o_{i}$ and $o_{j}$:

(13) $$d_{ij}=\mathrm{dist}\left(o_{i},o_{j}\right)$$

where dist(·,·) denotes the selected distance function, for which either Euclidean distance or cosine distance can be used. To distinguish between similar and dissimilar pairs, we define a binary adjacency matrix M, where $M_{ij}=1$ if $y_{i}=y_{j}$ (positive pair), and $M_{ij}=0$ otherwise (negative pair). The contrastive objective $L_{\mathrm{cont}}$ is formulated as the sum of the positive-pair loss $L_{\mathrm{pos}}$, which pulls samples from the same class closer together, and the negative-pair loss $L_{\mathrm{neg}}$, which pushes samples from different classes apart by at least a predefined margin *m*.

(14) $$L_{\mathrm{pos}}=\frac{1}{\sum_{i,j}M_{ij}}\sum_{i,j}M_{ij}d_{ij}$$

(15) $$L_{\mathrm{neg}}=\frac{1}{\sum_{i,j}\left(1-M_{ij}\right)}\sum_{i,j}\left(1-M_{ij}\right)\max\;\left(0,m-d_{ij}\right)$$

(16) $$L_{\mathrm{cont}}=L_{\mathrm{pos}}+L_{\mathrm{neg}}$$

#### 2.3.2. Classifier Loss

For the latent embedding O, we employ a deep classification head to map the embedding into the pan-cancer subtype label space y. The classifier is designed to capture high-order correlations within the latent embedding through a MLP architecture.

(17) $$\hat{y}=W^{\prime \prime }\left(\max\left(0,\mathrm{BN}\left(W^{\prime }O+b^{\prime }\right)\right)\right)+b^{\prime \prime }$$

where max(0,·) is ReLU activation function. The classifier is optimized using the cross-entropy loss [43]. Given the predicted $\hat{y}$ and the ground-truth y, the loss function is defined as:

(18) $$L_{\mathrm{cls}}\left(\hat{y},y\right)=-\sum_{c=1}^{C}y_{c}\log\left(\frac{\exp({\hat{y}}_{c})}{\sum_{j=1}^{C}\exp\left({\hat{y}}_{j}\right)}\right)$$

### 2.4. Biological Interpretability Assessment Methods

To examine whether the learned representations retained biologically relevant information, we analyzed the model at the input-feature, fusion, and latent-representation levels. Contributions of individual molecular features were quantified using Integrated Gradients (IG) [44]. For this analysis, 300 samples were randomly selected from those shared by all four omics modalities. IG was calculated for the ground-truth cancer-class logit using a zero baseline in the z-score-standardized input space and 20 interpolation steps. The absolute attribution values were averaged across samples, and duplicated features mapped to the same gene were represented by their maximum attribution. The top 200 genes from the gene-expression and mutation modalities were then examined for enrichment of a curated strict cancer-driver set [2] using an upper-tail hypergeometric test. Benjamini–Hochberg (BH) correction was applied to the enrichment tests.

To reduce the potential influence of mutation frequency on the driver-gene analysis, we further performed a frequency-matched perturbation experiment. In 1200 randomly selected samples shared by all four omics modalities, the standardized mutation inputs of 111 driver genes, including TP53, KRAS, PIK3CA, BRAF, VHL, IDH1, and ATRX, were set to zero, and the resulting decrease in the ground-truth class logit was calculated. The driver genes were divided into 10 bins according to their pan-cancer mutation frequencies. For each of 1000 repetitions, an equal number of non-driver genes was sampled without replacement from the corresponding frequency bins and perturbed in the same manner. The one-sided empirical *p*-value was calculated as the proportion of frequency-matched control sets producing a logit decrease at least as large as that of the driver-gene set, with an add-one correction. We also evaluated whether the classifier depended on molecular feature identity rather than only on the overall input magnitude. For 2000 samples proportionally stratified by cancer type, feature positions were independently permuted within each sample for each modality and for all four modalities together. This procedure preserved the within-sample value distribution and input magnitude while disrupting feature identity. Classification accuracy and the ground-truth class logit were summarized over five repetitions.

The feature gate and missing-modality mechanism were evaluated separately from the molecular-feature attribution analysis. In this study, modality-dependent input variation referred to candidate low-information features and differences in input magnitude or signal density that may affect representation learning. These features were used as operational statistical proxies and were not considered intrinsically biologically irrelevant. For a reproducible feature-level diagnostic, candidate low-information features were operationally defined as non-coding expression features identified from the feature names, mutation genes in the lowest frequency quartile, and methylation probes or CNV segments in the lowest variance quartile. Based on these operational groups, we assessed whether candidate low-information features were underrepresented among the top 200 features ranked by IG or gate-projection strength using lower-tail hypergeometric tests with BH correction. Gate-projection strength was calculated as the L2 norm of the projection weights associated with each input feature and was used to summarize the learned gating pattern. Because expression magnitude may affect attribution values, low-expression genes were additionally examined for overrepresentation using an upper-tail hypergeometric test. To evaluate the architectural effect of the feature gate, the trained sigmoid gate was compared with an all-ones gate and a fixed-random gate in 900 samples proportionally stratified by cancer type; classification accuracy, the ground-truth class logit, and cosine distance from the original embedding were recorded. Missing-modality behavior was assessed using the samples shared by all four modalities by removing every combination of one, two, or three modalities. Reconstruction of the removed modalities was evaluated using Pearson’s correlation coefficient, $R^{2}$, and the proportion of features with r>0.5. For single-modality removal, the change in the joint embedding was additionally measured by cosine distance. The four sample-specific MoE softmax weights were summarized across samples and cancer types, and the association between mutation-modality weight and the number of non-zero mutation indicators was evaluated using Spearman correlation. This quantity was treated as a mutation-count proxy rather than mutations per megabase.

Finally, we examined the associations between the joint embeddings learned by TMO-Net+ and known molecular states in the 12 cancer types used for the downstream prognosis task: BLCA, BRCA, CESC, GBM, HNSC, KIRC, LGG, LIHC, LUAD, LUSC, PAAD, and SARC. Within each cancer type, molecular states were defined using recognizable mutation patterns whose genes were present in the model input. Samples matching more than one state were considered ambiguous and excluded, and each retained group was required to contain at least 10 samples. Principal component analysis after feature standardization was used only for two-dimensional visualization. Silhouette coefficients [45] were calculated in the complete embedding space and evaluated using 1000 label permutations; non-positive coefficients were treated as lacking positive clustering evidence. Differences in the four MoE weights among molecular states were assessed using the two-sided Mann–Whitney *U* test for two groups or the Kruskal–Wallis test for more than two groups. BH correction was applied separately across the 12 silhouette tests and the 48 cancer-by-modality MoE tests. These analyses were used to assess whether the learned representations were consistent with established molecular characteristics within the TCGA cohort.

## 3. Dataset, Experimental Setting and Results

### 3.1. Dataset

The multi-omics dataset utilized in this study was sourced from a previous work [41] (available here: https://zenodo.org/records/11258239, accessed on 1 September 2026), which provides comprehensive multi-omics profiles for 32 cancer categories across 8238 samples from the TCGA database. After excluding entries with unspecified cancer categories, a final cohort of 8217 samples was retained for analysis. Each sample integrates four distinct omics modalities: RNA-Seq gene expression (6016 genes), DNA methylation (6617 probes), gene mutation (4539 genes), and copy number variation (CNV) (7460 segments). The foundation network initially undergoes supervised pre-training on this dataset to extract robust feature representations, which are subsequently transferred to three downstream clinical subtasks.

To evaluate the model’s cross-task transferability and predictive power, we conducted fine-tuning across three specific downstream subtasks: (1) Pan-cancer Classification: Multi-class discrimination among the 32 cancer types within the TCGA dataset. (2) Binary Metastasis Prediction: Assessment of tumor metastatic potential using an independent TCGA subset. (3) Prognostic Survival Prediction: Prognostic analysis focused on 12 cancer subtypes, adhering to a threshold of at least 50 uncensored samples per subtype to ensure statistical reliability. For the preprocessing of survival time (OS.time), missing or invalid entries are excluded from the survival analysis without imputation. Values less than 1 day are clipped to 1 day to ensure numerical stability, and all survival times are subsequently scaled by dividing by 30. The specific sample numbers for each cancer subtype are as follows: BRCA (757), LGG (497), LUAD (487), HNSC (486), LUSC (451), BLCA (400), KIRC (350), LIHC (345), CESC (275), SARC (229), PAAD (167), and GBM (118).

### 3.2. Experimental Setting

All experiments were performed on a workstation equipped with an Intel Core i5-13500HX CPU, 16 GB of RAM, and an NVIDIA GeForce RTX 4060 Laptop GPU, running on Windows 11 (version 24H2). The proposed method was implemented using PyTorch (v2.5.1) (https://pytorch.org/). To improve statistical reproducibility and account for variability introduced by stochastic model optimization, the main experiments were independently repeated five times using random seeds 66, 100, 166, 188, and 200. For each seed, all compared methods used identical training and test data partitions. All results are based on five independent runs and are reported with their corresponding 95% confidence intervals. Our experiments follow a two-stage process, encompassing the pre-training of the TMO-Net+ foundation model and its subsequent validation on three downstream subtasks.

Foundation Pre-training Stage: During the pre-training phase, the TMO-Net+ foundation model was trained on multi-omics data. Following prior work [41], for each omics, the encoder comprises two hidden layers with dimensions of 2048 and 512, respectively, while the decoder adopts a symmetric architecture with dimensions of 512 and 2048. The shared latent space is constrained to a dimensionality of 64. Omics and reconstruct discriminator are selected to improve the generative capacity of the foundation model. Both discriminators are composed of two linear layers with ReLU activation functions, each having a hidden layer dimension of 16. Their output dimensions correspond to the number of omics modalities (m=4) and a binary classification task (n=2), respectively. Training follows an alternating optimization strategy, in which the discriminators uses both the adversarial loss from the omics discriminators and discriminator loss as their total loss functions, and their parameters are updated before those of the main network. The total objective function of the main network comprises the self-modal ELBO from the self-VAE, the cross-modal ELBO from the cross-VAE, the K-L divergence between the original and inferred variational posteriors, the adversarial loss of discriminators, the contrastive loss, and the classifier loss. The main network is optimized using AdamW (learning rate $1\times 10^{-5}$, weight decay $1\times 10^{-5}$), whereas the discriminator-related modules use an independent Adam optimizer (learning rate $1\times 10^{-4}$). The learning rate is governed by a CosineAnnealingWarmRestarts scheduler with $T_{0}=10$, $T_{mult}=2$, and $\eta_{min}=1\times 10^{-6}$. With a batch size of 128, the main network is trained for a maximum of 300 epochs. Early stopping is implemented based on the LogME score of the validation set, with a patience of 10 and a minimum delta of 0.001.

Downstream Fine-tuning Stage: All downstream evaluations employed parameter transfer from the pretrained four-modality TMO-Net+ foundation model, followed by task-specific fine-tuning. To quantify the contribution of pretrained initialization in primary/metastatic prediction, an additional TMO-Net+ configuration was trained from random initialization using identical downstream data partitions. TMO-Net+ and TMO-Net+(pre) denote the randomly initialized and pretrained configurations, respectively, for the primary/metastatic prediction experiments. In the pan-cancer classification and prognosis prediction experiments, TMO-Net+ denotes the pretrained and subsequently fine-tuned configuration. (1) Pan-Cancer Classification: To classify the 32 cancer categories, we implemented a differential learning rate strategy: the encoder was assigned a learning rate of $1\times 10^{-5}$, while the classification head used $1\times 10^{-4}$. The learning rate was adjusted using the ReduceLROnPlateau scheduler (factor = 0.5, patience = 10). Early stopping was triggered based on the Macro-F1 score on the validation set (patience = 25). Final performance was evaluated on the test set using Accuracy, Macro-Precision, Macro-Recall, and Macro-F1. (2) Primary/Metastatic Prediction: For this binary classification task, we evaluated two configurations of the proposed model: TMO-Net+, which was trained from scratch on the downstream dataset, and TMO-Net+(pre), which was initialized using parameters transferred from the pretrained four-modality TMO-Net+ model. For TMO-Net+(pre), the GEX and methylation encoder parameters were initialized from the pretrained four-modality model; parameters of the auto-decoder, cross-decoder, shared encoder, and discriminator modules were also transferred when their dimensions were compatible, while all remaining parameters were randomly initialized. In contrast, all parameters of TMO-Net+ were randomly initialized and trained exclusively on the downstream training set. The model was optimized using AdamW (learning rate = $1\times 10^{-3}$, weight decay = $1\times 10^{-5}$) with a batch size of 64. Learning rate scheduling and early stopping (patience = 30) were governed by the ReduceLROnPlateau scheduler and validation Macro-F1 score, respectively. (3) Prognosis Survival Prediction: The model was trained using the Cox partial likelihood loss, integrated with a dynamically weighted reconstruction regularization term. The regularization weight, α, follows a piecewise linear scheduling strategy: it increases linearly from 0 to 1 during the initial 10 epochs and remains constant thereafter, denoted as α=min(1,epoch/10). Optimization was conducted using the Adam optimizer with a batch size of 128. Model performance was assessed via the C-index, which also served as the criterion for early stopping (patience = 40). To further evaluate clinical relevance, 5-fold cross-validation was implemented, with Kaplan-Meier (KM) survival curves generated by pooling predicted risk scores across all test folds for each cancer type. Patients were stratified into high- and low-risk groups based on the median pooled risk score, and the prognostic significance was assessed using the log-rank test to calculate *p*-values.

### 3.3. Ablation Experiments

To evaluate the contribution of each introduced component in the proposed model, we conducted extensive ablation studies using the original TMO-Net [41] as the baseline. For a rigorous comparison, all experiments were performed under a unified experimental setting on identical datasets, with LogME score [46] as the evaluation metric. As summarized in Table 1, the experimental results demonstrate the following: (1) Baseline Performance: The original TMO-Net achieves an overall LogME score of 1.0979, indicating its fundamental ability to extract discriminative latent representations from multi-omics data. (2) Individual Component Contribution: Integrating a Feature Attention mechanism into each omics encoder increased the score to 1.1480, indicating that attention-based weights effectively suppress redundant noise in high-dimensional features, thereby refining the quality of feature representation. The MoE module raises the performance to 1.1992, underscoring the role of expert-level nonlinear mapping in facilitating cross-modal integration; and incorporating supervised cancer category information via a classifier loss yielded a significant boost in the score to 1.3131, proving that task-specific supervision effectively guides the latent features toward a more separable and task-relevant distribution. (3) Synergistic Effects of Combined Modules: The integration of attention and MoE yields a score of 1.2019, exceeding the attention-only mechanism and aligning closely with the MoE-only variant, suggesting that MoE’s mapping further optimizes cross-modal integration once high-dimensional noise is mitigated. The combination of attention and supervised learning achieves a score of 1.3671, outperforming their individual applications, a gain attributed to simultaneous noise suppression and the infusion of categorical supervision. Similarly, the integration of MoE and supervised learning attains approximately the same score, surpassing their separate uses and demonstrating the strong complementarity between expert-based mapping and supervised information. (4) Complete Configuration: The complete TMO-Net+ configuration achieved the highest mean LogME score of 1.4202 among the evaluated variants, supporting the coordinated integration of the three introduced components.

### 3.4. Biological Interpretability Analysis

**Figure 2:**
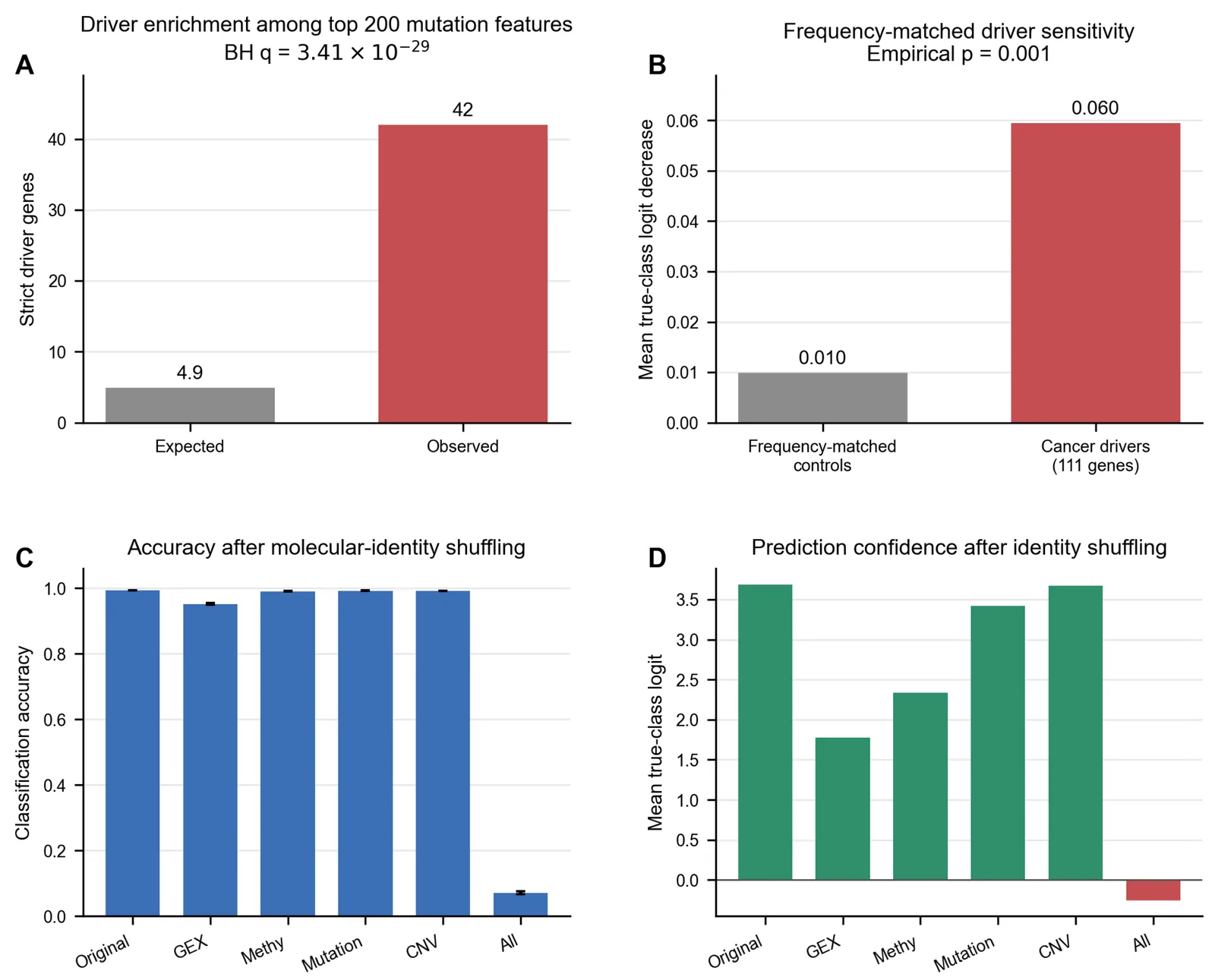
Biological relevance of input features and sensitivity to molecular identity. (**A**) The observed and expected numbers of cancer-driver genes among the top 200 mutation features ranked by IG. (**B**) Mean decrease in the ground-truth class logit after neutralizing 111 cancer-driver mutation inputs compared with mutation-frequency-matched non-driver controls. (**C**,**D**) Classification accuracy and mean ground-truth class logit after independently shuffling feature identities within each modality or across all four modalities. Error bars in panel C represent the standard deviation over five permutations. GEX, gene expression; Methy, DNA methylation; CNV, copy number variation.

Biological interpretability is important because improved downstream performance alone does not establish whether a model has learned meaningful molecular patterns. We therefore examined TMO-Net+ from the perspectives of molecular-feature importance, modality use, and cancer-specific molecular states. Rather than treating attention weights directly as biological importance [47], we first used IG to identify the input features that contributed to model predictions. Among the top 200 mutation features, 42 belonged to the strict cancer-driver set, whereas only 4.9 were expected according to their proportion in the background set (BH-adjusted $q=3.41\times 10^{-29}$; Figure 2A). These features included IDH1, BRAF, VHL, TP53, PIK3CA, KRAS, and ATRX, which represent well-established oncogenic, tumor-suppressive, and chromatin-regulatory events in different cancers. In contrast, only two strict driver genes occurred among the top 200 gene-expression features, and this enrichment was not significant (q=0.618). The correspondence with known cancer drivers was therefore most evident in the mutation attribution.

We next determined whether these driver genes were actually involved in the model prediction rather than merely appearing near the top of the attribution ranking. Setting the standardized mutation inputs of 111 driver genes to zero decreased the ground-truth class logit by 0.0595, compared with 0.00995 for mutation-frequency-matched non-driver genes (Figure 2B). None of the 1000 matched control sets produced a decrease as large as that observed for the driver genes (empirical p=0.001). This result shows that the model was more sensitive to known driver-gene identities and that the difference could not be explained only by their mutation frequencies. We further tested the importance of molecular identity by shuffling feature positions while retaining the values and overall magnitude of each sample. Shuffling gene-expression identities reduced classification accuracy from 0.9930 to 0.9522, whereas separately shuffling methylation, mutation, or CNV produced smaller changes (Figure 2C). When the identities of all four modalities were shuffled, accuracy decreased to 0.0713. The mean raw ground-truth class logit also decreased from 3.689 to 1.778 after gene-expression shuffling and from 3.689 to −0.255 after all four modalities were shuffled (Figure 2D). Thus, gene expression supplied the strongest individual identity signal, while successful classification depended on the coordinated identities of molecular features across the four modalities rather than on their aggregate input magnitude alone.

The feature-gate analysis further clarified how TMO-Net+ handled the different measurement characteristics of each omics modality. Candidate low-information features were absent from the top 200 mutation features ranked by IG, compared with a background proportion of 25.03%, and were also reduced in the methylation ranking (8.0% vs. 25.01%; both BH-adjusted $q<10^{-9}$). The gate-projection ranking showed a similar reduction in the predefined mutation, methylation, and CNV feature groups. However, the same pattern was not observed uniformly across all modalities. CNV features ranked by IG did not show significant depletion (q=0.103), while low-expression genes were enriched rather than depleted in the gene-expression ranking (39.5% vs. 25.0%, $p=3.05\times 10^{-6}$). A complementary gate-replacement analysis showed that replacing the trained gate with an all-ones gate left classification accuracy unchanged but altered the joint embedding (mean cosine distance from the original embedding, 0.0594), whereas a fixed-random gate reduced the mean ground-truth class logit by 1.486. Together, these findings indicate that low expression should not be equated with a lack of biological information and that the learned feature gate performed modality-dependent representation recalibration rather than applying a uniform filtering rule across all omics modalities.

At the modality level, the missing-modality and MoE analyses showed how complementary molecular information was combined. When one, two, and three modalities were removed, the mean Pearson correlations between the reconstructed and observed features were 0.711, 0.694, and 0.590, respectively. The corresponding mean $R^{2}$ values were 0.542, 0.517, and 0.414, while the proportions of reconstructed features with r>0.5 were 0.745, 0.744, and 0.580, respectively. Under single-modality removal, the mean cosine distance between the original and partial-omics joint embeddings ranged from 0.0018 to 0.0220 across the four removed modalities. The gradual decline indicates that the joint representation retained information shared across modalities, although recovery became more difficult as fewer observed modalities remained. The sample-specific MoE weights also changed with the molecular input profile. Across the complete cohort, mutation-modality weight was negatively correlated with the number of non-zero mutation indicators (Spearman ρ=−0.565), with stronger associations in SKCM (ρ=−0.911) and LUAD (ρ=−0.877). Samples with at least 300 non-zero mutation indicators received a lower median mutation weight than the remaining samples (0.212 vs. 0.439; Mann–Whitney $p<10^{-266}$). In other words, a dense mutation profile did not automatically dominate multi-omics fusion; its relative contribution decreased while the other modalities contributed more to the joint representation.

**Figure 3:**
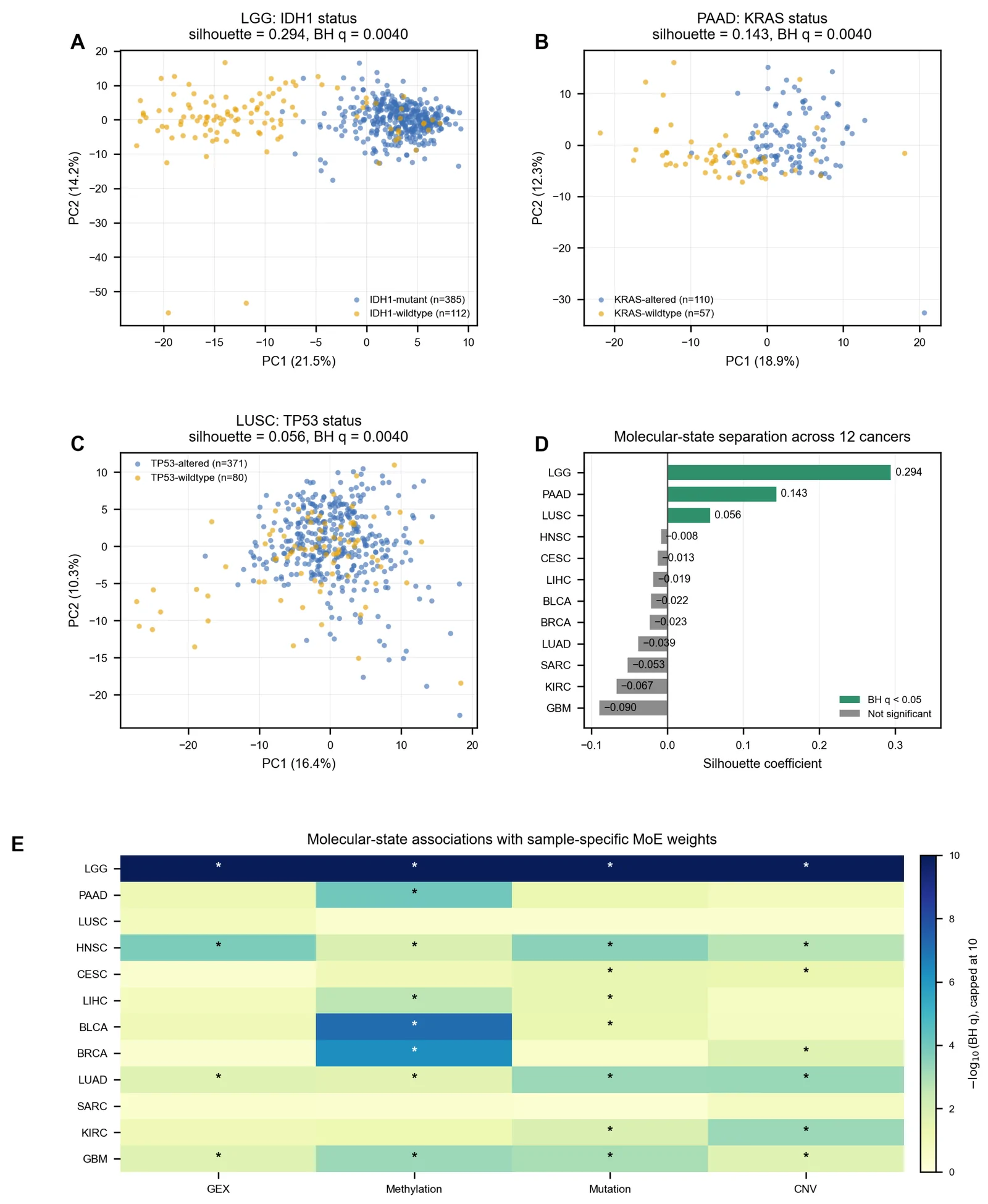
Cancer-specific molecular-state associations with the learned latent representations and MoE weights. (**A**–**C**) PCA projections of LGG according to IDH1 status, PAAD according to KRAS status, and LUSC according to TP53 status. (**D**) Silhouette coefficients for the mutation-defined molecular states across the 12 cancer types used in the prognosis task. The coefficients were calculated in the complete 256-dimensional representation. (**E**) Associations between molecular state and the four sample-specific MoE weights. Colors represent −$\log_{10}$-transformed BH-adjusted *q* values, capped at 10, and asterisks indicate q<0.05.

Finally, we asked whether the joint embeddings retained recognizable molecular organization within the 12 cancer types used in the prognosis task. LGG showed the clearest organization according to IDH1 status (silhouette coefficient, 0.294; Figure 3A). Most IDH1-mutant samples occupied a different region of the embedding space from IDH1-wildtype samples, consistent with the major molecular distinction associated with IDH1 in lower-grade glioma [48]. PAAD showed a more moderate separation according to KRAS status (0.143; Figure 3B), indicating that the representation retained information related to this central pancreatic cancer driver. LUSC showed only weak organization according to TP53 status (0.056; Figure 3C), with substantial overlap between the two groups. All three positive coefficients remained significant after permutation testing and BH correction (p=0.001, q=0.0040). The complete comparison in Figure 3D showed non-positive coefficients for the other nine cancer types. This does not mean that their embeddings lacked biological information; rather, the selected mutation states did not dominate the overall molecular heterogeneity represented by the model. The latent-space results therefore support cancer-dependent molecular organization, with the strongest evidence observed for IDH1-defined LGG.

The MoE results revealed a broader relationship with molecular state than the global embedding clusters. After BH correction, 10 of the 12 cancer types showed a significant difference in at least one modality weight among their molecular-state groups (Figure 3E). GBM, HNSC, LGG, and LUAD showed differences in all four modality weights, whereas LUSC and SARC showed no significant differences. Because the four softmax weights sum to one, these differences describe a relative redistribution of gene-expression, methylation, mutation, and CNV contributions. A molecular state may therefore change how TMO-Net+ combines the available omics information even when it does not produce a separate cluster in the final embedding. Taken together, the enrichment and perturbation of established driver genes, the dependence on molecular feature identities, the cancer-specific organization of the latent space, and the state-associated redistribution of modality weights provide complementary evidence that TMO-Net+ retained biologically relevant information at both the molecular-feature and multi-omics representation levels.

### 3.5. Performance Comparison on Downstream Pan-Cancer Classification

To evaluate performance on downstream pan-cancer classification, we conducted a comparative analysis between the baseline TMO-Net [41] and TMO-Net+ using Accuracy, Precision, Recall, and F1-score as evaluation metrics. As summarized in Table 2, TMO-Net+ consistently outperforms its baseline across all four metrics. Specifically, the mean accuracy and precision increased from 0.9627 and 0.9375 to 0.9689 and 0.9490, respectively, while the largest average improvements were observed in recall (from 0.9191 to 0.9444) and F1-score (from 0.9146 to 0.9443). These results indicate that TMO-Net+ extracts more discriminative pan-cancer feature representations from complex multi-omics data. In particular, the improvements in recall and F1-score suggest that TMO-Net+ reduces missed classifications and improves class-balanced performance.

### 3.6. Performance Comparison on Downstream Primary/Metastatic Prediction

To quantitatively evaluate the ability of our proposed TMO-Net+ model to distinguish primary versus metastatic cancer samples on downstream primary/metastatic prediction task, we have conducted extensive comparison experiments with mainstream models including SVM [49], CVAE [35], and TMO-Net [41]. Such experimental results are summarized in Table 3 on the metastatic dataset under unified settings, where the performance was evaluated using Accuracy, Precision, Recall, and F1-score.

As shown in Table 3, TMO-Net+ achieved an accuracy of 0.9272, precision of 0.9122, recall of 0.9193, and F1-score of 0.9140, whereas TMO-Net+(pre) achieved an accuracy of 0.9322, precision of 0.9249, recall of 0.9128, and F1-score of 0.9174. Compared with TMO-Net, both TMO-Net+ configurations achieved higher mean values across all four metrics. Among all evaluated methods, TMO-Net+(pre) achieved the highest mean accuracy, precision, and F1-score, whereas TMO-Net+ achieved the highest mean recall.Notably, the concurrent increases in Recall and F1 not only reflect improved overall correctness but also indicate clearer advantages in reducing missed detections and improving robust identification, thereby supporting more reliable prediction of primary/metastatic status.

These consistent gains align with the key design choices of the model: the VAE-based MoE fusion module dynamically integrates cross-modality information in the latent space and adaptively completes missing modalities. The gated MoE learns sample-dependent modality contribution weights, which helps enhance the separability of the joint embedding. And the feature attention encoder strengthens informative signals via feature gating in high-dimensional feature sequences, providing a more discriminative representation for primary/metastatic prediction.

### 3.7. Performance Comparison on Downstream Prognosis Prediction

To rigorously evaluate the efficacy and generalizability of the multi-omics latent representations learned by TMO-Net+ in downstream clinical prognostic tasks, we conducted extensive benchmarking across multiple cancer types within the TCGA pan-cancer dataset. TMO-Net+ was compared against contemporary state-of-the-art survival models, including PCLSurv [29] and MMOSurv [30], and the TMO-Net [41]. Performance was systematically assessed using the Concordance Index (C-index) with 95% confidence intervals, together with Kaplan–Meier (KM) survival analysis to evaluate its risk-stratification capability.

As summarized in Table 4, TMO-Net+ demonstrated competitive prognostic performance across the evaluated cancer cohorts. The overall average C-index increased from 0.6308 for the baseline TMO-Net to 0.6835 for TMO-Net+, which was also higher than those of PCLSurv (0.6405) and MMOSurv (0.6671). Notably, TMO-Net+ achieved the highest C-index in seven of the 12 cancer cohorts, including BLCA (0.6167), BRCA (0.7124), CESC (0.7593), HNSC (0.6132), LIHC (0.7123), LUAD (0.6711), and SARC (0.7858). These results underscore the capacity of our enhanced multi-omics architecture to capture and integrate complementary biological signals across diverse modalities.

TMO-Net+ did not achieve the highest C-index in GBM, KIRC, LGG, LUSC, and PAAD. In GBM, which contained only 118 samples, the relatively wide confidence intervals across the compared methods indicate greater uncertainty in performance estimation; therefore, the difference between TMO-Net+ (0.5951) and the highest point estimate obtained by TMO-Net (0.6357) should be interpreted cautiously. In KIRC and LGG, MMOSurv achieved the highest C-index, which may be related to its survival-specific few-shot meta-learning strategy and cross-cancer parameter initialization. Nevertheless, the difference between MMOSurv and TMO-Net+ was small in LGG (0.8551 versus 0.8472), with overlapping confidence intervals. In LUSC, PCLSurv achieved a higher C-index than TMO-Net+ (0.6422 versus 0.5936), possibly because its prototypical contrastive objective is specifically designed to improve the organization of survival-related representations. In PAAD, TMO-Net only slightly exceeded TMO-Net+ (0.6208 versus 0.6145), and their confidence intervals overlapped. These cancer-specific differences may therefore reflect cohort size, survival-event distribution, molecular heterogeneity, and the survival-specific optimization strategies used by the competing models. Unlike these task-specific survival models, TMO-Net+ is designed as a general multi-omics representation framework for multiple downstream tasks rather than being separately optimized for each cancer cohort.

**Figure 4:**
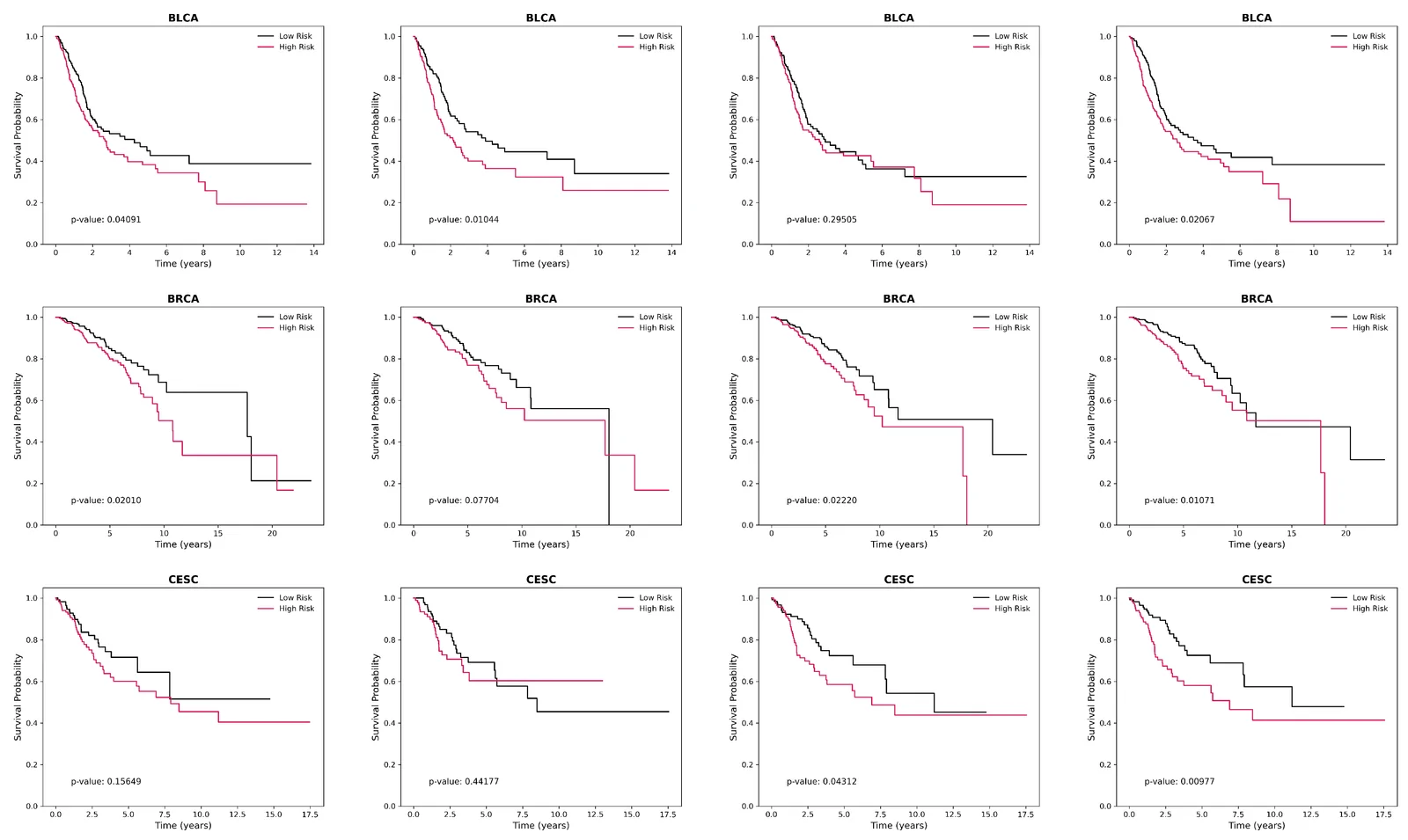

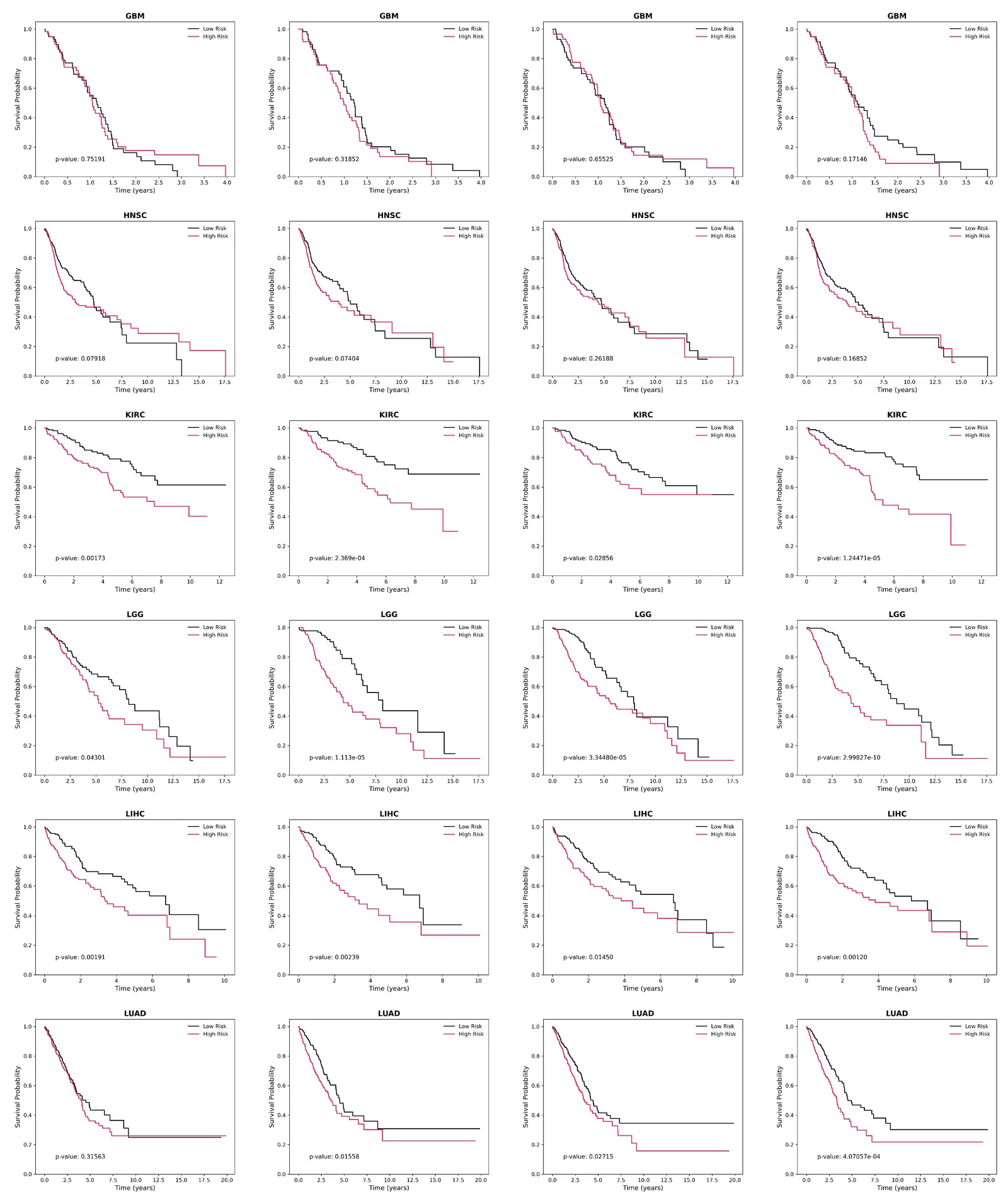

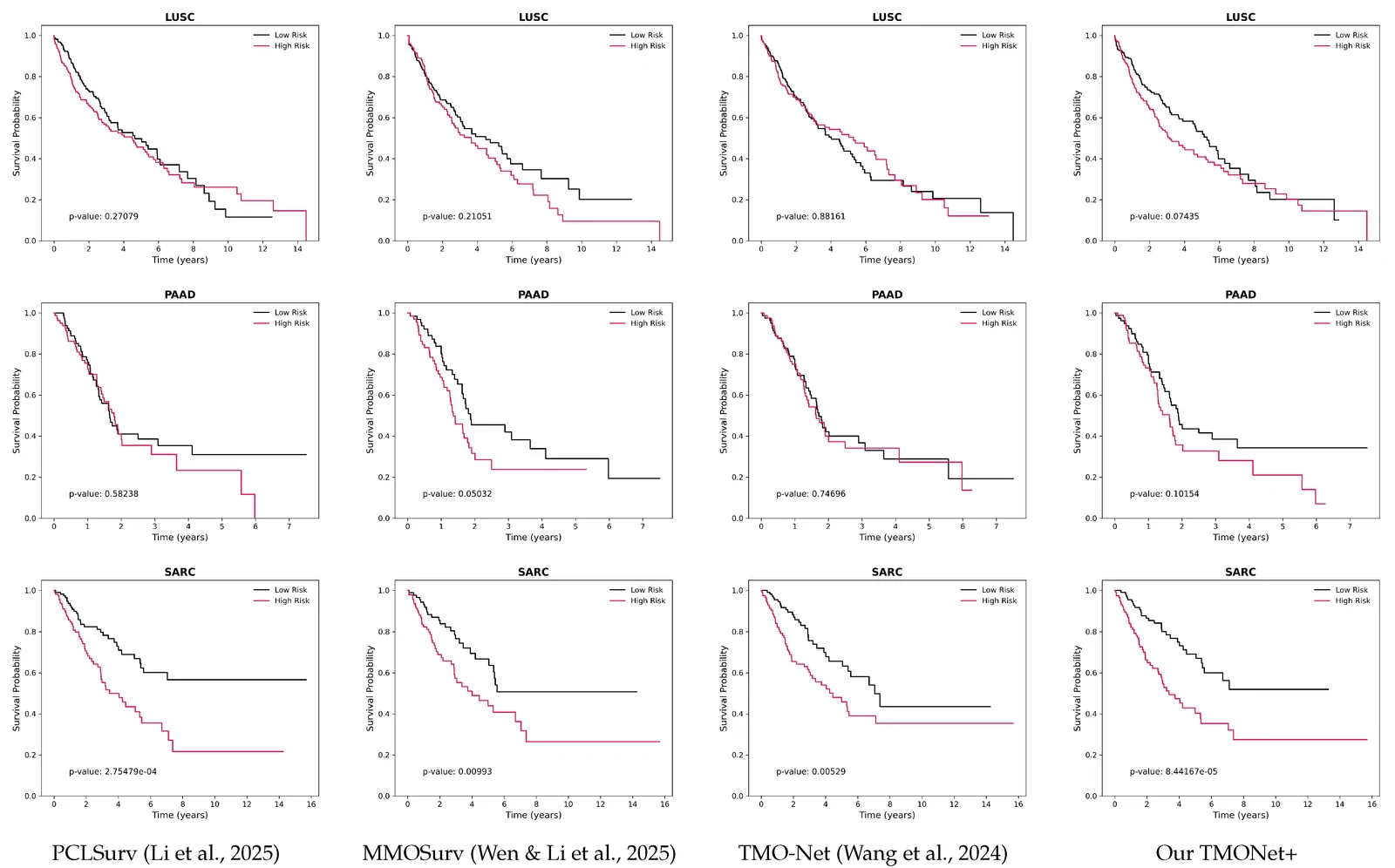
Kaplan–Meier analysis comparing overall survival prediction between baseline methods and the proposed TMO-Net+ model for stratifying patients into low-risk and high-risk groups across 12 cancer types [29,30,41].

At the level of clinical application, TMO-Net+ demonstrated profound translational utility in risk assessment. As illustrated by the Kaplan-Meier survival curves in Figure 4, risk scores derived from the model revealed distinct divergence in survival trajectories between high- and low-risk cohorts. Specifically, in key cancers such as BRCA, CESC, KIRC, LGG, and LIHC, the risk groups exhibited clear, non-overlapping, and statistically significant separation (Log-rank p<0.05 ). This confirms that the multi-omics representations extracted by TMO-Net+ possess not only high mathematical accuracy but also the ability to precisely characterize authentic biological prognostic variances, thereby providing a reliable decision-making foundation for personalized therapeutic strategies.

While specialized models such as MMOSurv and PCLSurv may exhibit localized advantages in Kaplan-Meier (KM) survival stratification (i.e., log-rank *p*-values) in selected cohorts, these localized performance gains are largely driven by their highly tailored optimization strategies. Specifically, MMOSurv employs a few-shot meta-learning strategy that uses cross-cancer parameter initialization to enable rapid task-level adaptation. This enables the model to establish stable risk-stratification boundaries in selected cohorts such as BLCA and PAAD, where MMOSurv achieved lower log-rank *p*-values than TMO-Net+ (0.01044 vs. 0.02067 in BLCA and 0.05032 vs. 0.10154 in PAAD), although the separation in PAAD remained borderline. Conversely, PCLSurv incorporates a prototypical contrastive learning mechanism that enforces representation consistency among samples of the same class, yielding a more compact risk-subgroup structure within the latent space. Such structural constraints may enhance the apparent separability of risk groups, translating to lower log-rank *p*-values in selected cohorts, particularly HNSC, where PCLSurv achieved a lower *p*-value than TMO-Net+ (0.07918 vs. 0.16852), although neither result reached statistical significance. In contrast, TMO-Net+ avoids artificially enforcing such discrete, clustered risk distributions, while achieving the lowest log-rank *p*-value in 8 of the 12 cancer cohorts and statistically significant risk-group separation in 8 cohorts. This cohort-specific variation helps explain why it may not invariably dominate in log-rank significance across all cohorts. Instead, TMO-Net+ is engineered to deliver a more balanced, robust, and formidable predictive capability across a broader pan-cancer landscape. By integrating a VAE-based MoE fusion module, it dynamically consolidates cross-modal information and adaptively compensates for missing modalities within the latent space. This mechanism facilitates the extraction of highly transferable, sample-level joint embeddings without relying on complex meta-learning paradigms or heavy dependence on class prototypes. Consequently, TMO-Net+ effectively captures the continuous nature of extreme intra-cancer heterogeneity, underscoring its substantial and reliable clinical value in multi-omics prognostic modeling.

## 4. Conclusions

To alleviate the pronounced heterogeneity and uneven modality quality issues present in tumor multi-omics data modeling, we proposed TMO-Net+, an enhanced pre-training framework. The framework introduces a synergistic combination of feature attention encoders to reduce the influence of modality-dependent input variation, a gated MoE mechanism coupled with a PoE strategy to capture sample-specific patterns while ensuring robustness against missing modalities, and a cross-task auxiliary supervision loss to improve embedding discriminability. Our experimental results demonstrate that TMO-Net+ learns sample-level joint embeddings that are transferable across the evaluated TCGA downstream tasks. Extensive benchmarking on pan-cancer datasets confirms that TMO-Net+ consistently outperforms existing methods across downstream tasks, including pan-cancer classification, primary/metastatic site prediction, and prognostic modeling, particularly in challenging modality-incomplete scenarios.

Biological interpretability analyses further showed that the learned representations prioritized established cancer-driver mutations and were sensitive to molecular feature identities. Under controlled modality removal, TMO-Net+ retained partially recoverable cross-omics information, while the latent embeddings and sample-specific MoE weights captured cancer-dependent molecular-state organization and relative shifts in modality contributions. Taken together, these findings support the internal biological consistency of the learned representations within the evaluated TCGA cohorts.

In the future, we will focus on incorporating more multi-omics clinical information and validating these biological associations in independent cohorts to better capture tumor heterogeneity and further enhance the model’s interpretability.

## Figures and Tables

**Table 1:** Performance comparison of different model configurations in the ablation study. LogME scores are reported as the mean and corresponding 95% confidence interval over five independent runs.

Methods	LogME Score
The Original TMO-Net [41]	1.0979±0.0769
+FeatureAttention	1.1480±0.0063
+MoE	1.1992±0.0252
+ClassifierLoss	1.3131±0.0122
+MoE+FeatureAttention	1.2019±0.0022
+ClassifierLoss+FeatureAttention	1.3671±0.0097
+MoE+ClassifierLoss	1.3676±0.0069
+ALL (Our TMO-Net+)	1.4202±0.0243

**Table 2:** Performance comparison between the baseline TMO-Net [41] and the proposed TMO-Net+ for pan-cancer classification.

Model	Accuracy	Precision	Recall	F1-Score
TMO-Net [41]	0.9627±0.0033	0.9375±0.0321	0.9191±0.0013	0.9146±0.0020
TMO-Net+	0.9689±0.0027	0.9490±0.0214	0.9444±0.0127	0.9443±0.0152

**Table 3:** Performance comparison of different methods under identical data partitions for primary/metastatic prediction.

Model	Accuracy	Precision	Recall	F1-Score
SVM [49]	0.7547±0.0105	0.8403±0.0077	0.8061±0.0097	0.8138±0.0061
CVAE [35]	0.8648±0.0266	0.8754±0.0248	0.8453±0.0237	0.8601±0.0207
TMO-Net [41]	0.8903±0.0273	0.8941±0.0201	0.8910±0.0198	0.8915±0.0123
TMO-Net+	0.9272±0.0188	0.9122±0.0258	0.9193±0.0243	0.9140±0.0219
TMO-Net+(pre)	0.9322±0.0272	0.9249±0.0307	0.9128±0.0404	0.9174±0.0339

**Table 4:** C-index comparison between the baselines and the proposed TMO-Net+ for prognosis prediction. Results are reported as mean ± 95% confidence interval.

	PCLSurv [29]	MMOSurv [30]	TMO-Net [41]	TMO-Net+
BLCA	0.5991 ± 0.0444	0.5932 ± 0.0406	0.5910 ± 0.1384	0.6167 ± 0.1138
BRCA	0.6551 ± 0.0364	0.6681 ± 0.0567	0.6407 ± 0.0881	0.7124 ± 0.0691
CESC	0.6181 ± 0.0767	0.6511 ± 0.1618	0.7454 ± 0.0866	0.7593 ± 0.0320
GBM	0.6277 ± 0.1110	0.6165 ± 0.2193	0.6357 ± 0.2246	0.5951 ± 0.1139
HNSC	0.6007 ± 0.0395	0.6101 ± 0.0652	0.5024 ± 0.0706	0.6132 ± 0.0706
KIRC	0.6949 ± 0.0367	0.7444 ± 0.0593	0.6551 ± 0.0580	0.6809 ± 0.0543
LGG	0.8542 ± 0.0471	0.8551 ± 0.0184	0.8194 ± 0.0889	0.8472 ± 0.0971
LIHC	0.6037 ± 0.0376	0.6779 ± 0.0432	0.6189 ± 0.2150	0.7123 ± 0.0734
LUAD	0.5876 ± 0.0484	0.6573 ± 0.0977	0.6070 ± 0.1683	0.6711 ± 0.1295
LUSC	0.6422 ± 0.0435	0.6276 ± 0.1742	0.5134 ± 0.0722	0.5936 ± 0.0689
PAAD	0.5786 ± 0.0462	0.6100 ± 0.2038	0.6208 ± 0.0939	0.6145 ± 0.0363
SARC	0.6241 ± 0.1180	0.6942 ± 0.1780	0.6204 ± 0.1868	0.7858 ± 0.0415
Average	0.6405 ± 0.0473	0.6671 ± 0.0464	0.6308 ± 0.0549	0.6835 ± 0.0524

## Data Availability

All data are from public datasets with download links given in the text https://www.kaggle.com/datasets/xiaozhuang1/tmonetplus (accessed on 1 September 2026), and the full code is available at https://github.com/xuanliu-nyist/TMONetPlus (accessed on 1 September 2026).

## References

[1] McGranahan N., Swanton C. (2017). Clonal heterogeneity and tumor evolution: Past, present, and the future. Cell.

[2] Martínez-Jiménez F., Muiños F., Sentís I., Deu-Pons J., Reyes-Salazar I., Arnedo-Pac C., Mularoni L., Pich O., Bonet J., Kranas H. (2020). A compendium of mutational cancer driver genes. Nat. Rev. Cancer.

[3] Weber L.L., El-Kebir M. (2021). Distinguishing linear and branched evolution given single-cell DNA sequencing data of tumors. Algorithms Mol. Biol..

[4] Weinstein J.N., Collisson E.A., Mills G.B., Shaw K.R., Ozenberger B.A., Ellrott K., Shmulevich I., Sander C., Stuart J.M. (2013). The cancer genome atlas pan-cancer analysis project. Nat. Genet..

[5] Liu J., Lichtenberg T., Hoadley K.A., Poisson L.M., Lazar A.J., Cherniack A.D., Kovatich A.J., Benz C.C., Levine D.A., Lee A.V. (2018). An integrated TCGA pan-cancer clinical data resource to drive high-quality survival outcome analytics. Cell.

[6] (2020). The ICGC/TCGA Pan-Cancer Analysis of Whole Genomes Consortium. Pan-cancer analysis of whole genomes. Nature.

[7] Chai H., Zhou X., Zhang Z., Rao J., Zhao H., Yang Y. (2021). Integrating multi-omics data through deep learning for accurate cancer prognosis prediction. Comput. Biol. Med..

[8] Boehm K.M., Khosravi P., Vanguri R., Gao J., Shah S.P. (2022). Harnessing multimodal data integration to advance precision oncology. Nat. Rev. Cancer.

[9] Guo F., Keese M., Zhao Y., Fu Q. (2026). Integrative Multi-Omics and Machine Learning Identify ID1 as a Candidate Gene Associated with Abdominal Aortic Aneurysm. Curr. Issues Mol. Biol..

[10] Raj A., Petreaca R.C., Mirzaei G. (2024). Multi-omics integration for liver cancer using regression analysis. Curr. Issues Mol. Biol..

[11] Tran K.A., Kondrashova O., Bradley A., Williams E.D., Pearson J.V., Waddell N. (2021). Deep learning in cancer diagnosis, prognosis and treatment selection. Genome Med..

[12] Krassowski M., Das V., Sahu S.K., Misra B.B. (2020). State of the field in multi-omics research: From computational needs to data mining and sharing. Front. Genet..

[13] Nguyen N.D., Huang J., Wang D. (2022). A deep manifold-regularized learning model for improving phenotype prediction from multi-modal data. Nat. Comput. Sci..

[14] Feldner-Busztin D., Firbas Nisantzis P., Edmunds S.J., Boza G., Racimo F., Gopalakrishnan S., Limborg M.T., Lahti L., de Polavieja G.G. (2023). Dealing with dimensionality: The application of machine learning to multi-omics data. Bioinformatics.

[15] Song M., Greenbaum J., Luttrell IV J., Zhou W., Wu C., Shen H., Gong P., Zhang C., Deng H.W. (2020). A review of integrative imputation for multi-omics datasets. Front. Genet..

[16] Flores J.E., Claborne D.M., Weller Z.D., Webb-Robertson B.J.M., Waters K.M., Bramer L.M. (2023). Missing data in multi-omics integration: Recent advances through artificial intelligence. Front. Artif. Intell..

[17] White I.R., Carlin J.B. (2010). Bias and efficiency of multiple imputation compared with complete-case analysis for missing covariate values. Stat. Med..

[18] Hassan A.M., Naeem S.M., Eldosoky M.A., Mabrouk M.S. (2025). Multi-omics-based machine learning for the subtype classification of breast cancer. Arab. J. Sci. Eng..

[19] Shen R., Olshen A.B., Ladanyi M. (2009). Integrative clustering of multiple genomic data types using a joint latent variable model with application to breast and lung cancer subtype analysis. Bioinformatics.

[20] Wang B., Mezlini A.M., Demir F., Fiume M., Tu Z., Brudno M., Haibe-Kains B., Goldenberg A. (2014). Similarity network fusion for aggregating data types on a genomic scale. Nat. Methods.

[21] Argelaguet R., Arnol D., Bredikhin D., Deloro Y., Velten B., Marioni J.C., Stegle O. (2020). MOFA+: A statistical framework for comprehensive integration of multi-modal single-cell data. Genome Biol..

[22] Hausmann F., Kurtz S. (2021). DeepGRP: Engineering a software tool for predicting genomic repetitive elements using Recurrent Neural Networks with attention. Algorithms Mol. Biol..

[23] Zhang X., Zhang J., Sun K., Yang X., Dai C., Guo Y. (2019). Integrated multi-omics analysis using variational autoencoders: Application to pan-cancer classification. Proceedings of the 2019 IEEE International Conference on Bioinformatics and Biomedicine (BIBM).

[24] Withnell E., Zhang X., Sun K., Guo Y. (2021). XOmiVAE: An interpretable deep learning model for cancer classification using high-dimensional omics data. Brief. Bioinform..

[25] Wang T., Shao W., Huang Z., Tang H., Zhang J., Ding Z., Huang K. (2021). MOGONET integrates multi-omics data using graph convolutional networks allowing patient classification and biomarker identification. Nat. Commun..

[26] Katzman J.L., Shaham U., Cloninger A., Bates J., Jiang T., Kluger Y. (2018). DeepSurv: Personalized treatment recommender system using a Cox proportional hazards deep neural network. BMC Med. Res. Methodol..

[27] Ching T., Zhu X., Garmire L.X. (2018). Cox-nnet: An artificial neural network method for prognosis prediction of high-throughput omics data. PLoS Comput. Biol..

[28] Huang Z., Zhan X., Xiang S., Johnson T.S., Helm B., Yu C.Y., Zhang J., Salama P., Rizkalla M., Han Z. (2019). SALMON: Survival analysis learning with multi-omics neural networks on breast cancer. Front. Genet..

[29] Li Z., Chen W., Zhong H., Liang C. (2025). PCLSurv: A prototypical contrastive learning-based multi-omics data integration model for cancer survival prediction. Brief. Bioinform..

[30] Wen G., Li L. (2025). MMOSurv: Meta-learning for few-shot survival analysis with multi-omics data. Bioinformatics.

[31] Chandrashekar P.B., Alatkar S., Wang J., Hoffman G.E., He C., Jin T., Khullar S., Bendl J., Fullard J.F., Roussos P. (2023). DeepGAMI: Deep biologically guided auxiliary learning for multimodal integration and imputation to improve genotype–phenotype prediction. Genome Med..

[32] Cohen Kalafut N., Huang X., Wang D. (2023). Joint variational autoencoders for multimodal imputation and embedding. Nat. Mach. Intell..

[33] Alatkar S.A., Wang D. (2023). CMOT: Cross-modality optimal transport for multimodal inference. Genome Biol..

[34] Zhao C., Liu A., Zhang X., Cao X., Ding Z., Sha Q., Shen H., Deng H.W., Zhou W. (2024). CLCLSA: Cross-omics linked embedding with contrastive learning and self attention for integration with incomplete multi-omics data. Comput. Biol. Med..

[35] Albaradei S., Napolitano F., Thafar M.A., Gojobori T., Essack M., Gao X. (2021). MetaCancer: A deep learning-based pan-cancer metastasis prediction model developed using multi-omics data. Comput. Struct. Biotechnol. J..

[36] Li R., Wu X., Li A., Wang M. (2022). HFBSurv: Hierarchical multimodal fusion with factorized bilinear models for cancer survival prediction. Bioinformatics.

[37] Oh J.H., Choi W., Ko E., Kang M., Tannenbaum A., Deasy J.O. (2021). PathCNN: Interpretable convolutional neural networks for survival prediction and pathway analysis applied to glioblastoma. Bioinformatics.

[38] Tang X., Zhang J., He Y., Zhang X., Lin Z., Partarrieu S., Hanna E.B., Ren Z., Shen H., Yang Y. (2023). Explainable multi-task learning for multi-modality biological data analysis. Nat. Commun..

[39] Moor M., Banerjee O., Abad Z.S.H., Krumholz H.M., Leskovec J., Topol E.J., Rajpurkar P. (2023). Foundation models for generalist medical artificial intelligence. Nature.

[40] Cui H., Wang C., Maan H., Pang K., Luo F., Duan N., Wang B. (2024). scGPT: Toward building a foundation model for single-cell multi-omics using generative AI. Nat. Methods.

[41] Wang F.a., Zhuang Z., Gao F., He R., Zhang S., Wang L., Liu J., Li Y. (2024). TMO-Net: An explainable pretrained multi-omics model for multi-task learning in oncology. Genome Biol..

[42] Chen T., Kornblith S., Norouzi M., Hinton G. (2020). A simple framework for contrastive learning of visual representations. Proceedings of the International Conference on Machine Learning.

[43] Mao A., Mohri M., Zhong Y. (2023). Cross-entropy loss functions: Theoretical analysis and applications. Proceedings of the International Conference on Machine Learning.

[44] Sundararajan M., Taly A., Yan Q. (2017). Axiomatic Attribution for Deep Networks. Proceedings of the 34th International Conference on Machine Learning.

[45] Rousseeuw P.J. (1987). Silhouettes: A Graphical Aid to the Interpretation and Validation of Cluster Analysis. J. Comput. Appl. Math..

[46] You K., Liu Y., Wang J., Long M. (2021). Logme: Practical assessment of pre-trained models for transfer learning. Proceedings of the International Conference on Machine Learning.

[47] Jain S., Wallace B.C. (2019). Attention is not Explanation. Proceedings of the NAACL-HLT 2019.

[48] (2015). The Cancer Genome Atlas Research Network. Comprehensive, Integrative Genomic Analysis of Diffuse Lower-Grade Gliomas. N. Engl. J. Med..

[49] He Y., Ma J., Ye X. (2017). A support vector machine classifier for the prediction of osteosarcoma metastasis with high accuracy. Int. J. Mol. Med..

